# Exploring residential care aide experiences with oral malodour in long‐term care

**DOI:** 10.1002/nop2.497

**Published:** 2020-05-12

**Authors:** Charanpreet Singh Dhami, Leeann R. Donnelly

**Affiliations:** ^1^ Department of Oral Biological and Medical Sciences Faculty of Dentistry The University of British Columbia Vancouver BC Canada

**Keywords:** healthcare assistants, malodour, nurses, nursing

## Abstract

**Aim:**

To determine what experiences do residential care aides (RCAs) have with individuals living with oral malodour in a long‐term care (LTC) facility?

**Design:**

Study investigation was done using an interpretive qualitative approach paired with a social constructivism interpretive framework.

**Methods:**

The study was conducted in May of 2015 through face‐to‐face interviews with RCAs, which were recorded and transcribed verbatim, observations of RCAs in their work environment, as well as RCA personal logs of their daily experiences with odour during caregiving. Thereafter, data were analysed and coded for emerging themes.

**Results:**

Five major themes were identified after grouping the codes: 1) attitudes and behaviours when caring for residents with malodour; 2)RCA knowledge of oral malodour; 3) level of job satisfaction among RCAs that care for residents with malodour; 4) culture and malodour; and 5) challenges of care giving compounded by malodour. These themes depict the overall experiences of study participants.

## 1 INTRODUCTION

The oral health status of geriatric people living in long‐term care (LTC) facilities is poor (Weening‐Verbree, Huisman‐de Waal, Dusseldorp, Achterberg, & Schoonhoven, 2013). This can be due to a decline in a resident's ability to perform personal oral care tasks and the reliance on others for assistance. Poor oral health conditions can contribute to oral malodour, a foul odour emanating from an individual's breath, which has a negative impact on an individual's social communication and social acceptability (Broek, Feenstra, & Baat, 2007). Evidence suggests that the geriatric population is more prone to oral malodour (Awano et al., 2011).

Residential care aides (RCAs) are an important component of the care facility team. They are frontline staff involved in the direct care of residents assisting them with their activities of daily living (ADLs) (McGilton, Sidani, Boscart, Guruge, & Brown, 2012). They also provide a valuable role in social and emotional support for residents. This becomes even more vital when residents have little interaction with family and friends, that leaves them emotionally vulnerable with a greater need to rely on their relationship with nursing staff to have a sense of self‐worth (Haugan, 2013). One of the many types of care RCAs provide daily is mouth care which includes brushing the resident's teeth, tongue and oral tissues and cleaning dentures. However, several studies have shown that daily mouth care is not carried out consistently or effectively in LTC facilities (Bowers, Esmond, & Jacobson, 2000; Dharamsi, Jivani, Dean, & Wyatt, 2009).

It is apparent that Western society places high importance on personal hygiene and grooming (Howson, 2013). Foul odour emanating from the body of an individual in old age is seen as a person in health decline, and it can limit meaningful interactions between a patient and their caregivers (Twigg, 2004). Much of what is known about oral malodour is related to sources, measurement techniques, management strategies and how malodour has an impact on the individual suffering from it ( McNab, 2008). However, we know little about how this condition has an impact on those around the sufferer and even less about how it has an impact on caregivers. Residents at LTC facilities can become very concerned about noticeable body‐related issues, such as malodour, because of the risk they feel of being avoided by other residents and staff (Donnelly, Clarke, Phinney, & MacEntee, 2015). Chronic oral malodour can affect approximately 20 to 50 per cent of the population (Nalini, Puneet, Kulmeet, & Kumar, 2011) Although residents have expressed concern over how they might be perceived and treated by caregivers due to this condition (Donnelly et al., 2015), it is unclear at this time if such concerns are valid, suggesting a need to better understand how resident oral malodour has an impact on RCAs in LTC facilities.

## 2 METHODS

An interpretive qualitative research approach was used in this study to explore RCA experiences with resident oral malodour to capture and describe their experiences when interacting with residents having oral malodour. Particularly, we were interested in what contributed to these experiences and how it affected the RCAs interaction with residents. Ethics approval for this study (H13‐0529) was received from the University of British Columbia, Behavioural Research Ethics Board.

The study was conducted using a social constructivist worldview, where multiple realities are constructed looking at the lived experiences of others (Creswell, 2013). The framework of social constructivism allows reality to be co‐constructed between the researcher and the participant and shaped by their individual experiences (Creswell, 2013). The goal of this research was to rely as much as possible on the views of RCAs when dealing with residents who have oral malodour. Yet, our own experiences were also used to analyse and interpret the information provided by RCAs.

### 2.1 Participant recruitment

Participants were recruited from one LTC facility located in Vancouver, British Columbia. This facility was selected for the study as oral malodour prevalence data from this site was available from a concurrent study. RCAs were recruited by holding an information session at the LTC facility. A total of 10 RCAs attended the session, at which time they were informed of the study and each given a consent form and a sample of the personal logs, which they would be required to complete as a participant in the study. Confidentiality was assured to the potential participants along with the option to withdraw from the study at their discretion. In total, seven RCAs participated in the study, six females and one male; this ratio is indicative of the gender distribution of RCAs at the LTC facility. Purposeful sampling was also used to recruit RCAs who were responsible for providing care to residents at a LTC facility who had already been assessed as having oral malodour. These participants were approached individually and given information on the study. The participants were required to have a minimum of one year of work experience to ensure that they had enough time to experience the central phenomenon and therefore able to provide valuable insight.

### 2.2 Observations

One week after the recruitment session, each participant was contacted and briefed on the study again and arrangements were made for one of the investigators (CD) to attend the facility to observe them in their daily activities and interactions with the residents. Observations were completed at various times during the day when care was being provided to residents. This variation allowed to document changes in workload for participants and changes in types of care provided to residents. Morning care is usually very busy for the participants, but also a time when odour is the strongest from residents as they are waking up to overnight incontinence pads needing to be changed and oral malodour experienced with morning breath. Approximately 15 hr were spent observing the seven participants. Prior to the observations, all the participants were informed that the researcher was simply present to observe and learn of their daily activities and to carry on with their duties as usual. The researcher always left the room if the resident did not give consent to be observed or had to be undressed to maintain dignity and respect for the resident. Each observation session lasted two to three hours, where one or two of the participants were shadowed providing care to various residents. Field notes were made of particular odours that were coming from the rooms of residents and how the participants dealt with these odours. Furthermore, body language and behaviours of participants were documented during their care with a particular focus on the provision of mouth care.

### 2.3 Personal Logs

Personal logs were provided to each participant on the day of their observation. Each participant was asked to complete 10 personal logs over a period of two workweeks. The purpose of the personal logs was to give participants the opportunity to document their thoughts and feelings about the odour experienced from residents during caregiving. The log questions were developed through literature review of caregiving in long‐term care and were designed to explore the experience the RCA had on that particular day caring for residents. On completion of the logs, they were collected, so that the information collected could aid in development of additional questions for the interview guide. This allowed the researcher to ask specific questions that could provide further clarification on individual and group comments contained in the logs, which helped gain a deeper understanding and meaning of their log statements.

### 2.4 Interview guide

The interview guide was developed from existing literature on caregiving in long‐term care, the participant's personal logs and through our observations. Statements made in personal logs, as well as researcher observations that needed further clarification, were added to the interview guide. The interview guide continued to evolve with more specific questions as more information was made available by participants in each successive interview. Face‐to‐face, audio‐recorded, personal interviews were conducted at the participant's convenience and lasted generally between 30–45 min. Following the interviews, each participant was given an honorarium for their participation in the study. Each was also informed that later the initial analysis would be shared with them so that they could review the findings and offer further insight or clarification to statements they may have made.

### 2.5 Data analysis

Each audio‐recording was transcribed verbatim following the interview. Four interviews were transcribed by one investigator (CD), and the other three were transcribed professionally and then reviewed by CD along with the audio‐recording to confirm accuracy of the transcription. Recordings and transcripts were listened to and read multiple times also by CD to become familiar with the data and to commence analysis. All observation notes, personal logs and interview transcripts were imported into the data management program N‐Vivo 10® and used in the analysis.

**FIGURE 1:**
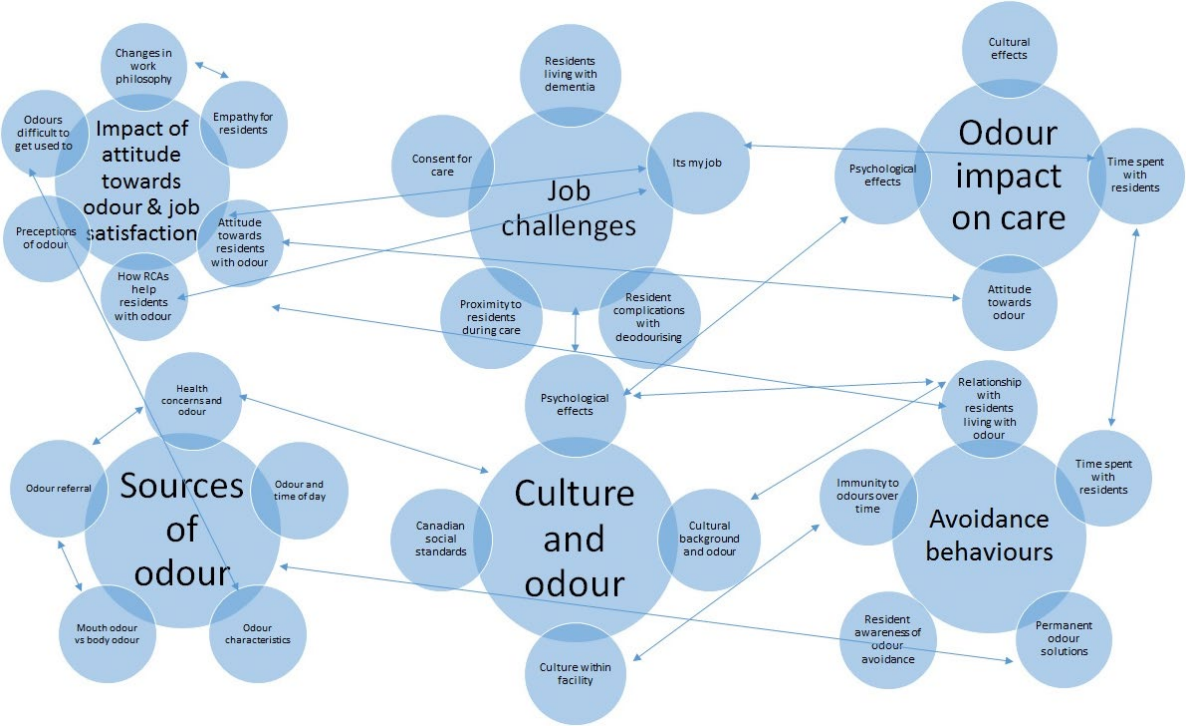
.Thematic Map: Visual map of grouping codes to form larger overarching themes

Codes were assigned to excerpts of the narratives, the observations and the comments made by participants in their personal logs. Coding was conducted by aggregating text data into small categories of information, seeking evidence for the code from different data sources used in the study and then assigning a label to the code (Creswell, 2013). On completion of this phase of the analysis, there were 54 codes that were then grouped and re‐arranged to form emerging themes. This was done by visually mapping the codes and looking at inter‐relationships. Please see Figure 1.

To ensure that the coding analysis was not biased, each author and two individuals with no dental background coded the first interview transcript separately and then compared their coding to look for similarities and differences. Overall, the coding analysis done by each party was quite similar with only slight differences that were discussed among the team to reach a consensus.

### 2.6 Rigour

There were several procedures adopted to establish rigour for this study. To gather candid responses from participants, confidentiality of participant responses was assured, and each participant was made aware that they had the opportunity to opt‐out of the study at any given point. Further, the research design had an observational component to ensure that statements made by participants were reflected by their actions. During and after data analysis, “member checks” were conducted with the participants face to face or via phone calls. Member checking refers to the participants being given an opportunity to verify statements made in the analysis to confirm whether what is recorded is actually what the participant was trying to convey (Shenton, 2004). This also allows participants the opportunity to provide further points of clarification on statements made and to verify accuracy of the analysis. All participants reviewed the initial analysis and were provided a list of their quotes that had been used. Three of the participants were asked for further clarification on statements they had made in their interviews. Using three different sources of data allowed the results to be confirmed by all three sources and added to the strength of this study.

## 3 RESULTS

Five major themes were identified after grouping the codes: 1) attitudes and behaviours when caring for residents with malodour; 2) RCA knowledge of oral malodour; 3) level of job satisfaction among RCAs that care for residents with malodour; 4) culture and malodour; and 5) challenges of care giving compounded by malodour. These themes depict the overall experiences our participants described when dealing with odour and, in particular, oral malodour, when working with their residents.

### 3.1 Attitudes and Behaviours when caring for residents with Malodour

Body odours in general were described by the participants as an unpleasant aspect of their job and in relation to that they also described several behavioural actions they would use to deal with this issue. These behavioural actions were mainly used to avoid the odour and usually resulted in what we observed as less than optimal care and social interaction. Participant two shared that she: “tries to get the job done as quickly as possible [laughter]” when helping residents with their daily care. She further elaborated by saying: “[odour] is really bad, you try to hold your breathe a little. Then the best thing to do is whatever's wet to get it out, tie it up or send it out of the room and continue doing [the care].” Participant four described her difficulty with a resident who had strong oral malodour: “this resident had such horrid breath it would be difficult to even enter the room.” This statement acknowledges the possibility that if the RCA chose to skip care on this resident it may go unnoticed given that this particular resident was quadriplegic, living with dementia and could not speak. Through our observations, we also noticed that the RCA participant and her teammate RCA for this particular resident worked quickly. Participant two shared further behavioural tendencies of dealing with odour: “that's why I carry perfume [laughter], or scented creams. I put it on my shirt so if [the odour] is really bad, then I just take a whiff of my clothes.” Participant one said: “if the resident has an [odour] problem sometimes we use the powder (laughing) to cover the smell. Powder works well to [mask] smell.” Participant four logged in her journal that: “sometimes I use a mask to protect myself from inhaling the odour, [and] I open windows slightly.” Opening windows was a very common odour avoidance behaviour that most participants admitted to using. None of the participants who indicated that they opened windows to avoid odour seemed to recognize, the negative impact on residents especially during colder weather when a cold breeze can be quite uncomfortable. Further, it appeared that residents were never asked for permission by the participant before these odour avoidance behaviours took place. Participant one shared a similar scenario:

One resident she always does her bowel movement in the morning… (Sigh) when I go inside the room I turn on the fan, [and] she always stops me! ‘No no no the fan is so loud, don’t turn it on.’ I say ‘no, I need the fresh air'. (laughing)

We also observed participant seven providing care to a resident who had significant oral malodour and it was quite obvious that she was keeping her distance from the resident. The participant informed me that sometimes she will give this particular resident orange juice because she believes it “freshens her breath.” Similarly, some participants described their use of mouthwash to lessen the malodour:

What I usually do is I try to… I don't know if it's right but if there's mouthwash, I mix it with the water. Let [the residents] gargle it first, spit it out before I actually brush their teeth. That's just my trick. That's how I do it, because if you just give them water, it's nothing. So at least it sort of masks the smell a little [laughter]. (Participant 2)

As a result of these avoidance behaviours, RCAs’ time spent with residents who have malodour is minimal. During our observations, we saw participants give residents quick wipes with wet towels for their bath and a quick wipe of their anterior teeth for mouth care.

Residents who are more cognitively aware do notice odour avoidance behaviour by RCAs. When we asked participants whether residents were aware of their odour avoidance behaviour, participant one shared: “of course they know… if somebody has no mental problem of course they know.” Participant four had a similar comment: “I think those people who still are functioning mentally, yeah they know of course they know, but I don’t [open windows] until they are out of the room.” Participant 2 discussed how she manages the situation: Some of [the residents] know. And then, some of them are like, ‘Why did you turn on the fan?’ and I say, ‘because it's hot in here,’…. I wouldn't say, ‘because you smell.’

This allowed the participant to both avoid the odour and at the same time avoid directly confronting the resident about the odour. Managing odour like this, from the participant’s perspective, causes no emotional harm to the resident.

### 3.2 RCA knowledge of oral malodour

Participants had difficulty describing what malodour smell was like, participant two commented: “For one resident, it's very sort of sour. Smelling like milk that's maybe soured”. Whereas participant four described it as: “very strong, sometimes it's like something rotten, it's in the air, like really rotten, like an egg, or garbage.” When I asked the participants what bothered them more mouth odour or body odour, most said mouth odour was more bothersome.

Most participants did not clearly understand oral malodour, all its sources and what they could do to eliminate it. During interviews, it was apparent that none of the participants had the same answer; some believed that oral malodour was a stomach issue; others believed it was due to tooth decay; and some did not have an answer. Most of the participants believed that malodour came from the stomach. This belief seemed to be related to their experience of oral malodour not always being resolved with mouth care. Participant six commented: “You know you clean the teeth [but I am not sure if odour] comes from the inside or what? (waving her hand from stomach up to the mouth) Like when they breathe out its [bothersome].”

Participant two had similar comments:

It's also what [residents] eat too. If they're people that love garlic, of course they're going to smell like garlic and medication too, I think it has an effect with their [stomach]. Sometimes if some residents have gastric problems and they're always burping, the gas is coming out, so [odour] is going to be coming out from their mouth.

Due to their beliefs on the sources of oral malodour, typically the referral to manage it goes to a nurse or physician. Participant four commented:

Usually, we report to the nurse in charge on that day and if the [odour] doesn't go away, like brushing doesn't help, then they put them on the doctor's list too; to check up why the odour is so strong?

While this would make perfect sense if the odour were coming from the stomach, few if any of the participants knew that nearly 90 per cent of malodour comes from the mouth and not the stomach indicating a referral is better suited for a dental professional who may better understand oral malodour and appropriate treatment options.

### 3.3 Level of job satisfaction for RCAs due to malodour

Some participants indicated that they measure their job satisfaction in part by whether they can help a resident eliminate odour through the care they provide. Albeit, some participants find odour difficult to deal with and this negatively affects their job satisfaction. When asked to describe their experience of providing care to those who have odour participant three responded:

my job is to eliminate it [odour] by giving them a good bath experience so that they're clean and healthy and I think maybe I feel bad for them. I feel like I want to help them be clean. So it doesn't bother me at all.

However, other participants described odour as a nuisance and an unfortunate part of working with residents. Participant one, when asked about her feelings on odour commented: “Of course I don't like it; you smell [odour] ooh! You want to run away.” When asked specifically about oral malodour from residents, she responded: “my reaction is ‘don't talk’ (laughs), ‘don't open your mouth’ (laughs).” This encouragement to not speak certainly seemed to undermine social relations between this RCA and the residents she worked with during our observations.

The participants that had a more positive attitude towards odour elimination tended to put resident care first and they showed more empathy, while participants who had a more negative view towards malodour tended to be more concerned about how odour affected them and made their job more difficult. Residents who were aware of RCAs using odour avoidance behaviours also have a greater chance of feeling insecure about themselves as a result decreased social interaction. When participant seven was asked about her interactions with residents who have oral malodour her response was:

I *would say it is minimal yes, we don’t want to interact too much, for example if* there are residents who are alone and want to talk. When we have time we go talk and spend time with them, but if there is more odour and even after doing your care and you know there is more [odour] it is to an extent minimized.

### 3.4 Culture and odour

Looking at the cultural background of the participants, there did not appear to be much difference in how they perceived odour; they all to a certain degree found it to be bothersome. Even when comparing how odour was viewed culturally where they grew up, some participants mentioned that they had more open space and did not sense too much odour from others. They did, however, admit that odour and being odour free is much more of a societal norm in Canada versus where they were raised whether it was the Philippines, Fiji or Hong Kong. Participant six shared: “I think in Canada, if you have body odour people look at you [funny] (laughing) but over [in the Philippines] people don't care.” What was particularly interesting was that some participants found it easier dealing with urine and faecal waste from residents than oral malodour. Participant five stated: “I still have to clean [the mouth] up because of the odour, the [bottom] side is better than odour coming from [the mouth]it smells…really I find it repulsive, but what can you do?…just put on a mask and go (laughing).”

There is also a workplace culture in the facility of how RCAs conduct themselves. The more experienced participants tend to mentor their newer co‐workers on how to deal with resident odours and odour avoidance techniques among the other aspects of long‐term care activities that they pass on. When asked about how she came up with the various odour avoidance techniques that she was using, participant two shared:

I guess it is trial and error and plus other staff members—because I haven't been working as a care‐aide that long. But then a lot of [care‐aides]—by observing them and by them telling me, ‘this works,’ or whatever, you just pick up what works best for you too.

### 3.5 Challenges of caregiving compounded by malodour

Participants detailed the numerous challenges they face daily when working with residents. One common challenge identified was the lack of time they have in providing care to residents. As participant one explained: “morning is very rushed, you have an hour and a half to get [residents] up, out, down [for breakfast].” Another shared:

you can't really do a good job if you're under these time constraints all the time. And I can imagine, the care aide's on the floor, they've got like 10 or 15 residents in the morning before breakfast. You try to do a good job. A job that you'd be proud of, I don't think you can do it. (Participant two)

To compound issues further, uncooperative, aggressive or combative residents can make caregiving even more difficult for participants. Given the time pressures, when a resident does not cooperate and/or has oral malodour, participants indicated a tendency to leave the resident alone or skip the daily mouth care so they can move on to the next resident. However, at some point, the resident will need care which can be difficult as participant four describes: “one elder punches and kicks when giving personal care and chews on the toothbrush. I try not to get close to the strong side of the elder to avoid being punched and kicked as much as possible. There is also a lot of verbal aggression as well.” Participant five added that with regard to providing mouth care: “sometimes [residents] spit at you or bite the toothbrush so what else can you do? You will do more damage, so I stop.” This was also observed when participant seven was working with a resident who had dementia and limited mobility. The participant commented during this observation that “she bites and swallows everything” so she simply wiped the residents front teeth with a damp cloth, leaving the back teeth untouched and the front teeth poorly cleaned. During this observation of participant, it was noted that she kept her distance from the resident and worked hastily as the malodour was clearly bothersome to her.

## 4 DISCUSSION

This study is the first that we know of to explore how odour and, in particular oral malodour of residents in LTC, might have an impact on RCAs and the care that they provide. Participant attitudes towards working with residents that were suffering from oral malodour were a key factor in determining the quality of care the resident received. Participants who showed empathy towards residents and who viewed oral malodour coming from residents as something that needed to be remedied through the care they provided, prided themselves on their ability to help the resident become odour free. When these participants perceived they were successful at eliminating odour they had, a more positive outlook on their work and seemed to show higher job satisfaction. In contrast, participants who viewed working with residents who have oral malodour, as a negative and nuisance, seemed to employ multiple odour avoidance behaviours. These avoidance behaviours lead to what can be considered substandard care and potentially contributed to strain on the resident and RCA interaction especially on those residents who were cognitively aware of their surroundings. We observed and heard from participants that their work environment is quite stressful at times due to being under strict time constraints and not having enough staff members to share their workload. Furthermore, participants who had uncooperative residents found providing care more challenging. Unfortunately, due to a lack of knowledge in oral malodour management and its sources and an apparent lack of belief in the efficacy of daily mouth care, participants were unable to understand that the same oral malodour that is bothersome to them on a daily basis can be reduced or at best eliminated by providing appropriate mouth care and referral for a dental evaluation. Doing this might end the continuous cycle of avoidance behaviours which can lead to emotional harm and social isolation of the resident.

Odour avoidance behaviours exhibited by participants for personal comfort can be harmful to residents either physically or emotionally and sometimes both. Similar types of odour avoidance behaviours have been observed among nurses dealing with dimethyl sulfoxide odour from patients receiving cancer therapy (Prior, Mitchell, Nebauer, & Smith, 2000). Nurses in these situations often worked as fast as possible while holding their breath, avoided speaking directly to patients or avoided them completely. Similarly, Dongen found nurses hold their breath and work as fast as they can when providing “bed and body work” (Dongen, 2001). Much of the literature that describes how odour has an impact on caregiving is in relation to body odours that are not associated with the mouth. However, the similarity in behaviours among participants in our study and more generally the health field indicate that whether the odour is originating from the mouth or from another body part, coping and adapting is required by caregivers.

How a caregiver copes and adapts to odour is influenced by their knowledge of the source and how to manage the problem. For the participants, it appeared that the lack of knowledge of oral malodour management strategies resulted in them developing avoidance behaviours, some of which were suggested by colleagues. Some participants commented that they tried to brush the residents’ teeth and still dealt with malodour postbrushing, it is unclear how well the mouth care was done. It is clear through literature that up to 90% of malodour is related to the mouth (Broek et al., 2007). Keeping the mouth clean through good thorough brushing should resolve malodour in most cases.

Further, attitudes towards working with residents with odour influenced the interaction between a resident and RCA. The participants employed many different odour avoidance behaviours, which they admitted could be obvious to residents, especially if they were cognitively intact. Most participants did not consult the residents when they used avoidance techniques, such as turning on fans or opening windows, which they felt did cause some tension. Such tension has been shown to cause residents’ to be less cooperative with their care, making the caregivers work more difficult, which could subsequently affect their morale and job satisfaction (McGilton et al., 2012). To improve RCA and resident relationship in this context, oral malodour management strategies should be included in RCA training programmes.

Positive caregiver approaches foster reciprocal resident behaviour that aids in improving their overall relationship and the psychological well‐being of both parties (Brownie & Nancarrow, 2013; McGilton et al., 2012). We did find some participants who genuinely wanted to help residents be clean and odour free and who viewed a pleasantly smelling resident as an indicator of a job well done, which lead to greater job satisfaction. Yet some of the participant's provided odour eliminating care out of an obligation to their job and did so quickly by giving residents a quick wipe under the armpits for bathing and a brush of a wet cloth across the front teeth for mouth care. Similar types of behaviours and attitudes have been observed with nurses in palliative care who also perform tasks such as mouth care which they describe as disgusting due to the odour, with care done out of obligation, as opposed to a caring attitude (Croyère, Belloir, Chantler, & McEwan, 2012). Others have found that RCAs do not like providing mouth care and see it as a repulsive task (Dharamsi et al., 2009). Regardless of whether this perspective is observed among nurses in palliative care or RCAs in LTC, this lack of a caring attitude coupled with obvious odour avoidance behaviours undoubtedly can affect the resident and caregiver interaction negatively.

Our findings are important because residents in LTC often rely on their caregivers not only for help with the ADLs but also for friendship and companionship (Hebert, 2010). When time spent by caregivers with a resident is minimal and task‐oriented, there is little potential for the residents to benefit psychologically from the interaction. Thus, when residents are asked, as one participant described, “not to talk,” or avoided completely because of mouth odour this can potentially have an impact on the resident emotionally. Residents rely greatly on their relationship with their caregivers for a sense of self‐worth, and when this relationship is poor, it can lead to depression and anxiety (Haugan, 2013). Others have also found that residents who have self‐perceived odours may also believe that they are negatively judged by caregivers, staff, visitors and other residents and tend to seek solitude to preserve personal dignity and avoid social embarrassment (Twigg, 2004).

To achieve a successful organizational culture for service delivery, a shared sense of responsibility and shared awareness of what improves residents’ quality of life is needed (Thorne, Kazanjian, & MacEntee, 2001). Furthermore, younger participants relied on their older, more senior colleagues for direction on how to best deal with situations involving body odour and oral malodour from residents. Unfortunately, due to an overall lack of knowledge of how to manage oral malodour, the information passed down to these younger, less experienced participants was avoidance behaviour techniques. Mentorship can be a positive experience, but when the mentor does not have adequate knowledge, this can lead to confusion and ambiguity for the mentee (Andrews & Wallis, 1999).

One of the emerging themes each of the participants wanted to discuss in this study was how a stressful environment coupled with time constraints affected their ability to care for residents. Rushed care due to time constraints can leave residents feeling as though they are part of an assembly line with little personal interaction. While we did not seek the resident's perception of their care in this study, others have found that this rushed behaviour can be very apparent and quite disturbing to residents (Donnelly, Clarke, Phinney, & MacEntee, 2016). The time constraints participants discussed are not unique as others have found similar complaints among nursing home staff (Testad, Mikkelsen, Ballard, & Aarsland, 2010; Tuckett et al., 2009). Increased control over workload and delivery of care are often suggested as strategies to improve job satisfaction, reduce caregiver burnout and staff turnover which can translate to improved health outcomes for residents (Testad et al., 2010; Wallin, Jakobsson, & Edberg, 2012). Time constraints coupled with a resident that exhibited oral malodour led to care that was visibly quite rushed.

To add to the existing stress of limited time RCA’s have to provide care, the issue was further compounded by aggressive and combative residents who made it difficult to provide care. Participants showed little interest in providing mouth care to individuals that were combative while they were observed. There also appeared to be a helpless attitude where participants felt that some situations were beyond their control. This is similar to findings from other studies on oral healthcare delivery in LTC facilities where combative and aggressive residents discourage caregivers from providing adequate mouth care, leaving staff frustrated and disengaged from the resident (Philip, Rogers, Kruger, & Tennant, 2012). Providing care to these type of individuals is difficult and when you have several more residents to see and you are at the same time smelling a foul order from the residents mouth, moving on to the next person appeared to be the easy option.

The LTC facility in this study promoted a person‐centred model of care through the Eden Alternative philosophy. Although we observed that the facility's physical environment ascribed to components of the philosophy, other aspects, such as shared decision‐making, placing the resident's preferences first and foremost and a non‐hierarchal workforce, were lacking. Participants often rushed care such as lifting residents out of their bed while they were still asleep to finish their job on time. This practice indicated that the resident's needs were not at the centre of the RCA’s decision‐making. This was also found by Ekman who saw care staff abandon person‐centred care when pressed for time to do the necessary work required of them (Ekman et al., 2011). However,such rushed care was not seen in the hallways and main gathering areas of the facility. Similarly, others who have looked at the efficacy of person‐centred care found that this was demonstrated when activities are visible to the public, as opposed to the care provided in bedrooms and bathrooms away from the public eye (Donnelly et al., 2015). We certainly observed definitive differences in the way that the study participants and some other staff interacted with the residents. Both the reported time challenges and disconnect between the different ways the staff appeared to have control over their work, suggested a lack of support from administrative staff. This lack of support for non‐hierarchal decision‐making among the staff and residents could be influencing the participants’ perceptions and our observations. Unfortunately, without support at all levels in the facility, implementation of a person‐centred model of care is not successful (Koren, 2010).

### 4.1 Limitations

There was only one male participant in our study and was due to the low number of male RCAs at the facility. Even though, the ratio of female to male participants is reflected by the facility's RCA workforce, having additional male participants would have increased the likelihood of more diverse male RCA experiences.

The interview sessions were allotted 60 min; however, most of the interview sessions lasted on average 30 min. This was primarily due to the language skills of participants where English was their second language, resulting in short concise answers. Also, the participants preferred to have their interview done during their lunch or dinner break as opposed to meeting after work hours. This arrangement might have led them to feel more rushed during the interview process as they were thinking about getting back to their work shift.

Language skills also had some impact on participant personal logs as the depth to which they were able to describe their experiences through written English language was limited. The researchers mitigated this shortfall by asking questions pertaining to their log entries during the interview process to ensure more accuracy and depth of statements made.

During observations, participants at first tended to be more cautious when providing mouth care possibly due to being observed by a dental hygienist. However, due to the time constraints RCAs face the participants soon tended to revert back to their regular practices; it is difficult to determine how direct observations had an impact on change in care.

## 5 CONCLUSION

Residents in LTC who are dependent on others for their care have a reasonable expectation to be treated with dignity and respect in a comfortable and caring environment that maintains their physical, psychological and social well‐being. Oral malodour among residents was a difficult condition for most of the caregivers in this study to deal with and appeared to have an impact on both the quality and quantity of care they provided. Numerous factors such as time constraints, inadequate staffing, combative residents and organizational culture of the facility also influenced the care provided by participants. Knowledge and understanding of the sources of oral malodour and how to manage it had a key influence on the provision of care among the participants. Many of the participants chose to use odour avoidance behaviours or avoid the resident altogether rather than focus on eliminating oral malodour or consult with a dental professional.

Thus, it is imperative that caregivers are informed of how they are able to help in resolving the ongoing cycle of oral malodour that leads to avoidance, which continually compounds the issue over time. If this realization is accomplished, it may help improve the caregiver and resident relationship and the overall well‐being of both.

## AUTHOR CONTRIBUTIONS

CD: Substantial contributions to conception and design, or acquisition of data, or analysis and interpretation of data. CD, LD: Drafting the manuscript or revising it critically for important intellectual content. CD, LD: Given final approval of the version to be published. Each author should have participated sufficiently in the work to take public responsibility for appropriate portions of the content. CD, LD: Agreed to be accountable for all aspects of the work in ensuring that questions related to the accuracy or integrity of any part of the work are appropriately investigated and resolved.
